# A Plea for Global Health Action Bottom-Up

**DOI:** 10.3389/fpubh.2016.00241

**Published:** 2016-10-31

**Authors:** Ulrich Laaser, Stephen Dorey, Joanna Nurse

**Affiliations:** ^1^Section of International Public Health (S-IPH), Faculty of Health Sciences, University of Bielefeld, Bielefeld, Germany; ^2^Commonwealth Secretariat Health and Education Unit, London, UK

**Keywords:** global threats, public health professionals, global health action, bottom-up initiatives

## Abstract

This opinion piece focuses on global health action by hands-on bottom-up practice: initiation of an organizational framework and securing financial efficiency are – however – essential, both clearly a domain of well-trained public health professionals. Examples of action are cited in the four main areas of global threats: planetary climate change, global divides and inequity, global insecurity and violent conflicts, and global instability and financial crises. In conclusion, a stable health systems policy framework would greatly enhance success. However, such organizational framework dries out if not linked to public debates channeling fresh thoughts and controversial proposals: the structural stabilization is essential but has to serve not to dominate bottom-up activities. In other words, a horizontal management is required, a balanced equilibrium between bottom-up initiative and top-down support. Last but not least, rewarding voluntary and charity work by public acknowledgment is essential.

## Introduction

The plea for Good Global Governance, published recently (1) as predominantly top-down political strategy for global health, needs to be complemented by a bottom-up strategy to gain momentum. Can public health professionals contribute to initiate and sustain bottom-up initiatives coping with the main global threats as there are planetary climate change, global divides and inequity, global insecurity and violent conflicts, and global instability and financial crises? Can they advance global health as a human right for all?

Richard Horton, editor of the Lancet, recently addressed the Arab public health community referencing the violent conflicts in the Arab world and terrorist bloodshed in Europe (2): “What can the public health community in the Arab World, together with your friends internationally, do to address these crises? This may seem a hopelessly naive question. We are merely public health professionals not politicians, and certainly not politicians with power, but I truly believe that public health, in its best tradition, can have (even should have) a response.” Part of a response has been cited in the Skopje Declaration of the Public Health Collaboration Network in South Eastern Europe (PH-SEE) in 2002 (3): “Public health professionals promote peace and tolerance through education of health professionals, service to society, and research into health disturbance from conflict, poverty, and vulnerability of the population.” In this “perspective article,” we advance in more detail possible roles of public health professionals in bottom-up initiatives.

## The Role of Public Health Professionals

By referring back to the global threats listed in the plea for Good Global Governance (1), we discuss examples of bottom-up activities which require nevertheless overarching support in two ways to be sustainable: first, initiation of an organizational framework and second, financial efficiency. Both components are required for lasting success and both require skills public health practitioners possess including epidemiology (to identify threats), management (to organize the control of threats), health promotion and prevention (to contain the threats), and environmental health (to build healthy conditions necessary for well-being) (4).

### Component (1) Initiation of an Organizational Framework

Many western countries have a long and sustainable tradition of self-help groups for various conditions. In the example of Germany below, this has been developed further and since the mid-1980s into a broader movement (5), e.g., in 1992, the German federal state of North Rhine-Westphalia being among the first to set up a collaborative overarching support structure for bottom up initiatives within the framework of the WHO Regions for Health Network (RHN) (6): establishment of annual health conferences at the communal as well as at the state level (7, 8) with the participation, e.g., of medical and pharmacists chambers, hospitals, health and social insurances, employers, trade unions, and self-help associations. These structures have addressed topics that include developing psychiatric care outside the hospital, improving health promotion and self help, improvement of citizen and patient participation in health care, or the development of multi-media in the health care system.

### Component (2) Securing Financial Efficiency

To grow and sustain a bottom-up initiative, and preserve autonomy a secure financial investment is beneficial, regardless of whether this is based primarily on small voluntary contributions from participants or from larger non-governmental or governmental donors. In order to attract investment, it is important to prove effectiveness of proposed activities by robust evaluation and dissemination through media and health conferences to demonstrate that invested resources are being utilized in not just the most effective but also the most cost-effective way (9). A multilateral approach to financial support is often more resilient and sustainable, including state support combined with charity foundations and, where appropriate, private investment. Political decision makers have recognized the need to include non-governmental funding in their social development initiatives and can facilitate this through tax incentives and other measures such as advocacy. Three factors we see as conditional in securing this governmental support:

(a) Informed public debate on disadvantaged or vulnerable group.
(b) Repositioning of the secular ethical norms of solidarity and equity in the society.
(c) Public and systematic acknowledgment of voluntary work and charity.

## Action on Global Health Threats

In the context of the two components outlined above, how could public health professionals support action in the social and health arena on each of the four global threats?

### Planetary Climate Change

Central to the 2030 Agenda on Sustainable Development (10) is ensuring sustainability of the natural environment alongside meeting the wider needs of society to protect and promote societal and individual well-being (11). It is self-evident that fundamental to human well-being is a well-functioning ecosystem and biosphere. Conversely, maintaining a healthy environment and ensuring the transition to environmental sustainability requires human societies that function well (12).

With reference to the climate summit in Paris (2015), the World Federation of Public Health Associations (WFPHA), a non-governmental organization (NGO), implemented a survey to evaluate the actions of national governments in protecting the health of their citizens from the impacts of climate change (13). National public health associations, medical associations, and other health professional organizations responded, providing information on the actions of 35 governments spread across the globe. The survey revealed a severe lack of climate health preparedness with one third of participating countries indicating no national plan to protect citizens from the health impacts of climate change. One of the key recommendations asks “That WFPHA members and other health and medical professional associations around the world continue to make it a priority to raise the awareness of all national governments as to the multiple risks, and vulnerabilities for health and well-being from climate change, the opportunities for health co-benefits associated with climate change mitigation and adaptation, and the need to prioritize public health within their mitigation and adaptation policy, planning and programs.”

One of the challenges of tackling climate change has been the perception that it is a problem for the environmental community to tackle and that, though important, never at the top of the list of priorities for other sectors such as finance and international trade. More effectively demonstrating the clear and present threat to society could be a very effective way of shifting this focus. Public health professionals have a central role in here by advocating the links with health and well-being of humans as well as other organisms and the wider environment. Promoting both the “One health” and “Planetary health” agendas would be a good example of this (14).

### Global Divides and Inequity

Globally, the gap between the very rich and the poor has been widening at least for the last two decades. The reasons for this ever widening inequality are complex, often occurring perversely, for example, due to capital having a higher accumulation rate than income. Fundamental structural measures will need to be found to tackle this at source such as global tax on capital (15). However, these are complex, even “wicked” (16), problems and will take time to solve; in the meantime, the world is confronted with the consequences: armed conflicts due to poverty and lack of development, and resulting waves of migration. The majority of NGOs act on the spot, dealing with the consequences. However, migrants arriving in new countries need language training, counseling, and schooling for their children to prevent them slipping in to poverty in the new communities they arrive in. For example, in Germany, up to 15% of new migrants live below the poverty line, which is only likely to be stirring up further social problems for the future. A very impressive example of a bottom-up initiative aiming to tackle this is the so-called Spendenparlament (Sponsoring Parliament), in the Internet (17). Founded in 1996 by citizens in Hamburg, there are now over 20 similar initiatives across central Europe. These parliaments have conducted open sessions in the Internet raising more than 10 million EURO and supporting over 1100 projects to tackle poverty, homelessness, and isolation through social projects in the local community. The principles of the Sponsoring Parliament can be summarized as follows:

- Independent;
- Funding only of sustainable projects;
- Funding only once;
- Tax deductible;
- Public decision-making on funding;
- The decisions are binding;
- Passive membership possible;
- In honorary capacity only.

Examples of projects from the 62nd session in 2016 are

*Theaterkurse für junge unbegleitete Flüchtlinge* (theater courses for unaccompanied refugee children): 2016, 62. Session; subsidy amount: 6.000 Euro.
*Psychologische Betreuung traumatisierter Flüchtlingsmädchen* (psychological counseling for traumatized refugee girls): 2016, 62. Session; subsidy amount: 28.234 Euro.
*Aerztliche und soziale Beratung für Menschen ohne Papiere* (medical and social counseling for people without documents): 2016, 62. Session; subsidy amount: 72.000 Euro.

### Global Insecurity and Violent Conflicts

Worldwide, armed conflicts are responsible for more casualties than any single disease as well as wasting resources directed toward building and distributing armaments that could be otherwise allocated to population health and well-being and social development rather than death and destruction. The challenge to eliminate armed conflicts and their death toll is immense but so are the rewards possibly leading to alternative scenarios as a piece of thought (18).

**Table:** 

Scenario	Strategy
1.Armed conflicts between and within countries remain largely uncontrolled	1.The present political and organizational arrangements are replaced by effective consensus building and reconciliation
2.The use of military force becomes a sole prerogative of the United Nations (Security Council)	2.The military–industrial complex is dissolved
3.Technological advance allows for use of force without taking lives	3.The underlying reasons of armed conflict are removed (i.e., poverty, hunger, humiliation, and lack of education)

For the time being, we are left with the first scenario and the third strategy to remove the underlying reasons for armed conflict. Geoffrey Rose, the late English cardiologist and epidemiologist, insisted that the tail belongs to the body of a distribution, i.e., extreme phenomena are determined by the average experience (19): the general level of aggressiveness in a society is related to the phenomenon of extreme violence. Health professionals can make a threefold contribution to violence prevention: (1) analyze the causal interrelationships of violent phenomena; (2) curb the underlying reasons or determinants of violence by advocating implementation of evidence based approaches; and (3) train the workforce for this increasingly important aspect of protecting and promoting health. One example of this is the installment of so-called “hate-watching” groups to monitor and evaluate the early signs of public intolerance or speech of hate targeting minority groups. Historical examples of public language change set a worrying precedent such as are 1930s Germany and 1980s Yugoslavia and well as worrying trends in contemporary society (20–22).

### Global Instability and Financial Crises

The threats described above have progressed to such a degree that the current political, social, and economic order of the world is threatened. Instability is increasing including in the case of the Middle East across whole regions. This is happening alongside other worrying trends such as gross deficits in the implementation of Development Assistance for Health (DAH) [according to Ref. (23)]:

The overwhelming temptation to accept international aid without conditions on the side of the beneficiary often disrupts national priorities particularly if money comes too easily without sufficient checks. Loans for example of the World Bank – though at low interest rates – often put a great burden on later years, promoting intergenerational inequality and threatening longer term economic sustainability. Loans have two sides: Money is available now but has to be repaid later (especially if by others – taxpayers in the next generation). In addition, large portions of the lent money can be lost into expert fees and purchase of equipment, often back to the crediting countries rather than benefiting the recipient economy. The resulting question is rarely asked: Is the long-term outcome worth the (national) investment? The answer depends also on the structural sustainability of projects, which in the majority of projects is impaired by the limited funding perspective of 2 or 3 years and disconnection of potential follow-up activity.

A considerable share of DAH is channeled through NGOs. Transaction costs are high demonstrating the need for an external integrated tracking system (24). A code of conduct on cooperation with governments for national and international NGOs and a type of accreditation procedure at least at the national level is required to improve global governance. There is also a need to develop greater alignment and coordination between NGOs themselves to improve efficiency and prevent unproductive competition and duplication of effort. Bjegovic-Mikanovic et al. have assembled a list of global health networks including many NGOs (25).

## Conclusion

Initiation of the two components, first, an organizational framework and second, secure financial efficiency are domains where trained public health professionals have the necessary skills to make valuable contributions. Their participation in postgraduate programs and continued education (25) is essential for an organized effort to cope with the global threats discussed here. However, the focus of their training has to be shifted from epidemiology to health management, i.e., from analysis to intervention. Often we re-confirm what we know already. Likewise, service capacity has to be re-allocated and resources be joined through efficient multi-professional networking.

Activities as described in this paper at the local, regional, or global level are always at risk of fading away before being fully completed and effective or of remaining unknown without the potential to serve as useful a model elsewhere in the world. Therefore, a stable health systems policy framework (26) would greatly enhance success. Health conferences organized and supported by the respective governmental structures constitute one model proven to be feasible. Another option can be the long-term support by a larger foundation or a contractual agreement between several partners/institutions. The key condition is that interests are neutralized and in any case made public and transparent. Sometimes, it may be difficult to find a satisfying solution here. On the other hand, such organizational framework dries out if not linked to public debates channeling fresh thoughts and controversial proposals: structural stabilization is essential, but it is important that this exists to serve and not dominate the essential bottom-up activities. In other words, a horizontal management approach (7) is required, achieving a balanced equilibrium between bottom-up initiative and top-down support. Initial public debate can be triggered by asking for the limitations of utilitarian ethics discussing efficiency (27). Last but not least, rewarding voluntary and charity work with public acknowledgment is essential to encourage others to join in.

## Author Contributions

UL drafted this paper. SD checked the language and commented on selected issues. JN revised the first draft and advised on the second one.

## Conflict of Interest Statement

The authors declare that the research was conducted in the absence of any commercial or financial relationships that could be construed as a potential conflict of interest.
